# Isorhamnetin inhibited the proliferation and metastasis of androgen-independent prostate cancer cells by targeting the mitochondrion-dependent intrinsic apoptotic and PI3K/Akt/mTOR pathway

**DOI:** 10.1042/BSR20192826

**Published:** 2020-03-18

**Authors:** Fangzhen Cai, Yanmei Zhang, Jianwei Li, Sihuai Huang, Ruilin Gao

**Affiliations:** Department of Urology, The Second Affiliated Hospital of Fu Jian Medical University, Quanzhou 362000, China

**Keywords:** Apoptosis, Isorhamnetin, Metastasis, PI3K/Akt/mTOR, Prostate cancer

## Abstract

The present study investigated the effects of Isorhamnetin on two types of prostate cancer cells (androgen-independent and androgen-dependent) and explored its possible mechanisms underlying such effects. Treatment with Isorhamnetin significantly inhibited cell growth and induced lactate dehydrogenase (LDH) release of androgen-independent DU145 and PC3 prostate cancer cells, but exhibited almost no toxicity effect on androgen-dependent LNCaP prostate cancer cell line or normal human prostate epithelial PrEC cells, which was achieved by the induction of apoptosis in a mitochondrion-dependent intrinsic apoptotic pathway. Furthermore, Isorhamnetin inhibited cell migration and invasion in concentration-dependent manners by enhancing mesenchymal−epithelial transition (MET) and inhibiting matrix metalloproteinase (MMP) 2 (MMP-2) and MMP-9 overexpression. In addition, Isorhamnetin also down-regulated the expression of phosphorylated PI3K (p-P13K), Akt (p-Akt), and mTOR (p-mTOR) proteins in both cancer cells, revealing Isorhamnetin to be a selective PI3K–Akt–mTOR pathway inhibitor. In summary, these findings propose that Isorhamnetin might be a novel therapeutic candidate for the treatment of androgen-independent prostate cancer.

## Introduction

Prostate cancer represents as one of the most commonly diagnosed cancer in the United States and remains the second leading cause of cancer deaths in men, trailing only lung cancer [1,2]. Strong evidence suggests prostate cancer cells cannot grow or differentiate without androgens, therefore, androgen ablation therapy can be considered as one of the preventive approaches employed to manage prostate cancers [3]. Though this therapy is effective, most of these patients inevitably established the resistance to androgen deprivation, developing into androgen-independent prostate cancer [4]. These tumors are highly aggressive, more resistant to currently used chemotherapeutic agents, and more likely to metastasize from the primary site to distant tissues than other tumor types [5,6]. Therefore, targeting androgen-insensitive prostate cancer cells is an important strategy in drug discovery efforts, despite current taxane or provenge-based drugs, which are the only chemotherapies approved for this condition by the United States Food and Drug Administration (FDA) in that survival benefits are modest [7]. Taking into consideration that chemotherapy has severe side effects and usually a poor outcome, there is a growing demand for the development of safer and more therapeutic agents to improve the treatment outcomes of hormone-refractory prostate cancer.

Recently, great attention has been paid to the components obtained from natural source, due to their potential tumor selectivity, cytotoxic efficacy, and few side effects. Numerous *in vitro* studies have been performed to identify the potent chemopreventive naturally occurring compounds to treat and prevent this malignancy [8,9]. In this context, developing novel anticancer agents from traditional Chinese medicinal herbs which offer significant protection against the development of human prostate cancer is highly desirable. Isorhamnetin (3′–methoxy–3,4′,5,7–tetrahydroxyflavone) is a flavonoid isolated from traditional Chinese medicine such as *Ginkgo biloba, Persicaria thunbergii* H., and *Hippophae rhamnoides* L. [10,11] As an immediate metabolite of quercetin, it has been considered as an anticancer agent against a wide range of cancers, including esophageal and gastric cancer, leukemia, skin, colon, and lung cancer [12], and in most cases, it induces higher cytotoxicity toward tumor cells than quercetin [13]. Despite this background, to the best of our knowledge, there is lack of information available to describe the antitumor potential of isorhamnetin on androgen-independent prostate cancer cells and the mechanisms underlying these effects remain unclear. Currently, there is a growing recognition that the PI3K/AKT/mTOR pathway emerges as a distinct intracellular signaling pathway in driving prostate cancer cells resistance to androgen deprivation therapy and triggering tumor progress in the setting of castrated levels of testosterone [14,15], which is deregulated in 42% of locally advanced prostate cancers and nearly 100% of advanced prostate cancers [16,17]. Our preliminary *in vitro* assay showed that isorhamnetin can impede the Akt activity in androgen-independen prostate cancer cells. It was possible that antitumor effect of isorhamnetin on androgen-insensitive prostate cancer is achieved by suppressing the PI3K-Akt–mTOR pathway. Therefore, the aim of the present study was to evaluate the effect of the profile of isorhamnetin against two different human prostate cancer cells cultured *in vitro* and validate if this specific mechanism is involved in this cell death.

## Materials and methods

### Materials and reagents

**Figure 1:**
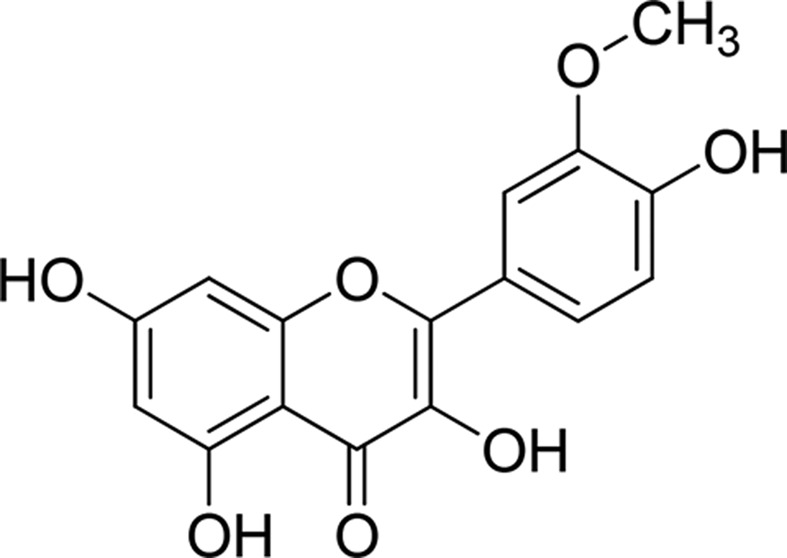
Chemical structure of isorhamnetin

Isorhamnetin (3′–methoxy–3,4′,5,7–tetrahydroxyflavone; Figure 1) with a purity of up to 98% was purchased from Sigma–Aldrich (St. Louis, MO, U.S.A.). Dulbecco’s modified Eagle’s medium (DMEM) and fetal bovine serum (FBS) were purchased from Invitrogen Co. (Grand Island, NY, U.S.A.). 3-(4,5-dimethylthiazol-2-yl)-2,5-diphenyltetrazolium bromide (MTT) and Annexin V- Fluorescein Isothiocyanate (FITC) kit was procured from BD Biosciences (San Diego, CA, U.S.A.). Monoclonal antibodies against Bax, Bcl-2, cytoplasmic cytochrome-*c*, cleaved-caspase 9, cleaved-caspase 3, cleaved-PARP protein, E-cadherin, Vimentin, N-cadherin, matrix metalloproteinase (MMP) 2 (MMP-2), MMP-9, phosphor(p)-PI3K (p85), PI3K (p85), p-Akt (phosphorylated Akt) (Thr^308^), Akt (Thr^308^), phosphorylated mTOR (p-mTOR), mTOR, and β-actin were purchased from Santa Cruz Biotechnology, Inc. (Santa Cruz, CA, U.S.A.). The Boyden chamber was purchased from Neuro Probe Inc. (Gaithersburg, MD, U.S.A.). The lactate dehydrogenase (LDH) assay kit was from Nanjing Jiancheng Bioengineering Institute (Nanjing, China). The BCA Protein Assay kit, enhanced chemiluminescence (ECL) detection system, and horseradish peroxidase–conjugated secondary antibody were obtained from Pierce (Rockford, IL, U.S.A.). All other reagents and chemicals used in experiment were from Sigma–Aldrich (St. Louis, U.S.A.).

### Cell lines and cell culture

Androgen-independent DU145 and PC3 cell line and androgen-dependent LNCaP cell line were purchased from the Cell Bank of Type Culture Collection of Chinese Academy of Sciences (Shanghai, China), and were cultured in DMEM supplemented with 10% heat-inactivated FBS, penicillin (10 U/ml) and streptomycin (10 μg/ml) in a humidified incubator (37°C, 5% CO_2_). Normal human prostate epithelial cell line PrEC were cultured in RPMI 1640 medium under the same conditions. All cells were harvested after a brief incubation in 0.25% (w/v) ethylenediaminetetraacetic acid (EDTA) in phosphate-buffered saline (PBS), followed by trypsinization (0.25% w/v trypsin/0.53 mmol/l EDTA in PBS).

### MTT assay

MTT assay was used to evaluate the effect of Isorhamnetin on the cell viability of different cancers and normal cells and was performed as described previously [18]. This colorimetric assay is based on the reduction in yellow MTT to blue formazan by mitochondrial dehydrogenase, which reflects mitochondrial functions and hence cell’s viability. The absorbance value of formazan is directly proportional to the number of viable cells [19]. Briefly, cells were plated at a density of 4 × 10^3^ cells/well in 96-well plates with 100-μl cell culture medium and incubated at 37°C for 24 h. Cells were subsequently treated with Isorhamnetin (0, 2.5, 5, 10, and 20 μM) for 24, 48, and 72 h. Following the treatments, the original medium was replaced with fresh culture medium containing MTT (0.5 mg/ml) and then, cells were incubated for an additional 4 h. At the end of experiment, the formazan product formed by MTT was dissolved by DMSO and the optical density (OD) was read at 570 nm wavelength in a microplate reader (Bio-Rad, Hercules, CA). The assays were performed in triplicate in three independent studies and the percentage of viability was calculated according to the formula: % viability = [mean OD treated cells × 100]/(mean OD control cells).

### Measurement of LDH release

The release of LDH from cells was used to investigate the cell toxicity. Briefly, different cells were seeded at 2 × 10^5^ cells/well in six-well plate and allowed to adhere overnight. Next, cells were treated with Isorhamnetin at various concentrations (5, 10, and 20 μM) for 48 h. Following centrifugation at 10000×***g*** for 10 min, the LDH release from cells into medium was measured by the LDH detection kit according to the manufacturer’s protocol.

### Apoptosis analysis by flow cytometry

An Annexin V-FITC Apoptosis Detection Kit was utilized to measure the percentage of apoptosis in cancer cells following different treatments. Briefly, after treatment with Isorhamnetin at indicated time period in six-well microplates, the cells were harvested, washed, and transferred to flow cytometry tubes in 500 μl of 1× binding buffer, followed by the addition of 5 μl of Annexin V–FITC and 5 μl Propidium Iodide (PI) for 5 min in the dark at room temperature according to the manufacturer’s protocol. Apoptotic cells were analyzed by FACS Calibur Flow Cytometer with CellQuest Pro software (Becton Dickinson, San Jose, CA).

### Boyden chamber invasion and migration assay

The Boyden chamber was used to evaluate the effect of Isorhamnetin on cell invasion and migration ability of cancer cells as described by Yang et al. [20]. After treatment for 48 h, cells were detached by trypsin, resuspended in serum-free DMEM, and loaded on to the upper compartment of the Boyden chamber at a density of 10^4^ cells/well. For invasion assay, polyvinyl-pyrrolidone-free polycarbonate filters (8-μm pore size) were precoated with the reconstituted basement membrane Matrigel (50 μg/filter) and the lower chambers were filled with DMEM containing 10% FBS as a chemoattractant. After incubation at 37°C in a humidified incubator for 24 h, the floating cells on the upper surface of the membrane were carefully removed with a cotton swab, while other cells on the lower filter surface were was fixed with 100% methanol, stained with 0.5% Crystal Violet, and counted under a light microscope. For migration assay, no coating of Matrigel on polycarbonate filters and all procedures were performed in the same conditions as above. Each experiment was performed in triplicates. Invasion and migration values were expressed as means ± SD of the percentage of the number of invaded or migrated cells relative to control from three independent experiments, each carried out in duplicate.

### Western blotting analysis

After treatment, the cells were harvested, washed, and solubilized in RIPA lysis buffer to extract total cellular proteins. The supernatant was collected by centrifugation at 12000×***g*** for 10 min and stored at −70°C until use. The protein concentrations were determined by a BCA Protein Assay kit as per the manufacturer’s instructions. Each equal amount of protein (40 μg) was added to 12% sodium dodecyl sulfate/polyacrylamide gel electrophoresis gel (SDS/PAGE), and transferred on to polyvinylidene difluoride (PVDF) membrane, which was then blocked with 5% non–fat milk in Tris–buffered saline and Tween 20 (TBST) buffer for 1 h. After blocking, the membrane was incubated with a 1:1000 dilution of particular primary antibody for Bax, Bcl-2, cytoplasmic cytochrome-*c*, cleaved-caspase 9, cleaved-caspase 3, cleaved-PARP protein, E-cadherin, Vimentin, N-cadherin, MMP-2, MMP-9, phosphorylated PI3K (p-PI3K), PI3K, p-Akt (Thr^308^), Akt, p-mTOR, mTOR, and β-actin at 4°C overnight and then blotted with the appropriate horseradish peroxidase-linked secondary antibody. After washing three times, bound antibodies on the membrane was visualized using an ECL detection kit (Amersham Biosciences, GE Healthcare, U.K.) and the band intensity of β-actin was used as an internal control.

### Statistical analysis

All values were expressed as mean ± S.D. of three independent experiments. The statistical significance was performed using the two-tailed Student’s *t* test or one-way ANOVA. The *P*-value of less than 0.05 was considered significantly different.

## Results

### Isorhamnetin induces growth suppression and LDH release of androgen-independent DU145 and PC3 cells, but not androgen-dependent LNCaP cells

**Figure 2:**
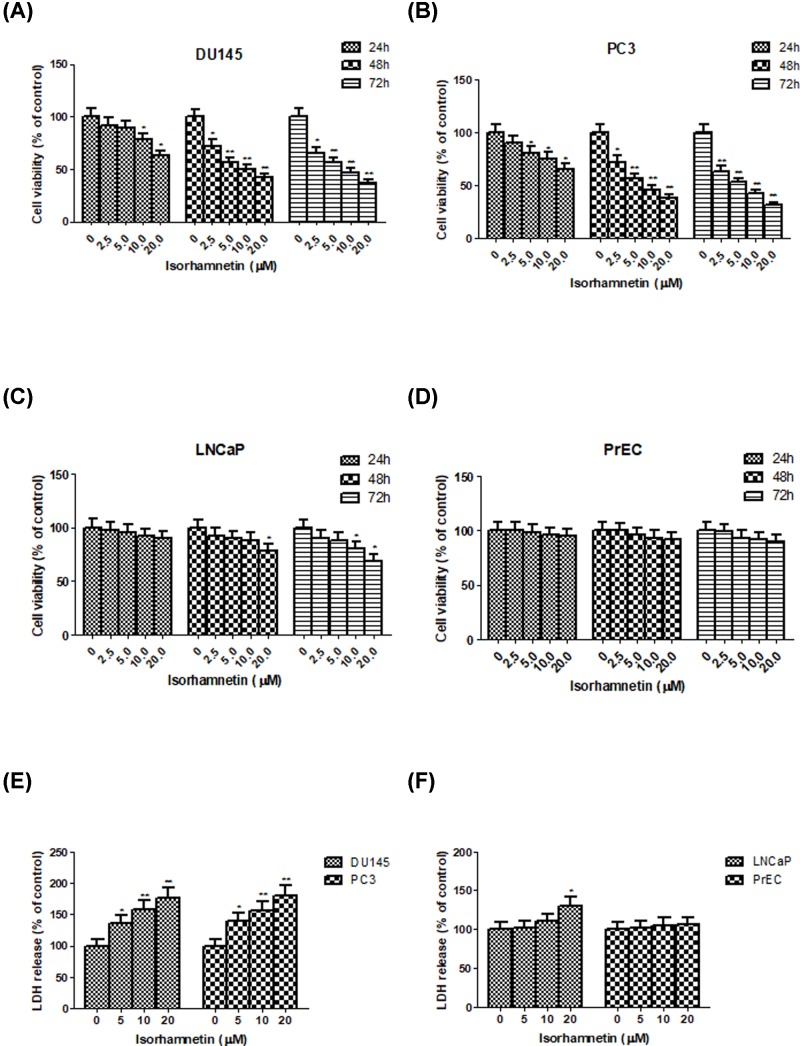
Isorhamnetin inhibits cell growth and induces LDH release in prostate cancer cell lines (**A**) Effects of Isorhamnetin (2.5, 5, 10, and 20 μM) on the cell growth of DU145 cells. (**B**) Effects of Isorhamnetin (2.5, 5, 10, and 20 μM) on the cell growth of PC3 cells. (**C**) Effects of Isorhamnetin (2.5, 5, 10, and 20 μM) on the cell growth of LNCaP cells. (**D**) Effects of Isorhamnetin (2.5, 5, 10, and 20 μM) on the cell growth of PrEC cells. (**E**) Effects of Isorhamnetin (5, 10, and 20 μM) on the LDH release of DU145 and PC3 cells. (**F**) Effects of Isorhamnetin (5, 10, and 20 μM) on the LDH release of LNCaP and PrEC cells. Related data appear as the means ± SD of three separate assays. *P<0.05 and **P<0.01 vs control.

To explore the cytotoxicity of Isorhamnetin on various human prostate cancer cells, androgen-independent cell lines (DU145 and PC3) and androgen-dependent cell line (LNCaP) were treated with increasing concentrations of Isorhamnetin (0.5–20 μM) or vehicle for 24, 48, and 72 h, and then analyzed by MTT assay. Statistical analyses between control and treatment groups revealed significant growth inhibition in androgen-independent DU145 (Figure 2A) and PC3 cell lines (Figure 2B) following treatment with Isorhamnetin in comparison with the controls (*P*<0.05 or *P*<0.01). Moreover, such suppression was in a concentration- and time-dependent manner. The inhibitory effect of Isorhamnetin toward both cells at the concentration beyond 2.5 μM and the time period at 48 h was approaching that at 72 h under the same conditions. However, significantly decreased cell viability in androgen-dependent LNCaP cell line occurred only when the concentration is 20 μM at 48 h and beyond 10 μM at 72 h as compared with the untreated control (*P*<0.05, Figure 2C). More importantly, no toxicity was observed in normal human prostate epithelial cell line PrEC in the presence of Isorhamnetin at all time (Figure 2D). Hence, androgen-independent human prostate cancer DU145 and PC3 cells were more sensitive to growth inhibition by Isorhamnetin compared with androgen-dependent LNCaP cells. Based on the above results, for convenient and efficient use, we chose the concentrations of 5, 10, and 20 μM, and the incubation time of 48 h as optimal parameters in the further experiments for studying the antitumor activity of Isorhamnetin on DU145 and PC3 cells.

The release of LDH from cells into the extracellular medium is one of the indicators that are often used to evaluate the effect of a drug to cause cell death. As shown in Figure 2E, after treatment with Isorhamnetin (5, 10, and 20 μM) for 48 h, the release rate of LDH was obviously increased in a dose-dependent manner in androgen-independent DU145 and PC3 cells compared with the control group (*P*<0.05 or *P*<0.01), but keep almost no change for androgen-dependent LNCaP cell line (a light significant difference from control at 20 μM of Isorhamnetina, *P*<0.05) and normal human prostate epithelial PrEC cells (Figure 2F). These results clearly indicated that Isorhamnetinat causes the release of LDH to promote cell death in androgen-independent DU145 and PC3 cells.

### Isorhamnetin promotes apoptosis and alters the expression of apoptosis-related protein of androgen-independent DU145 and PC3 cells

**Figure 3:**
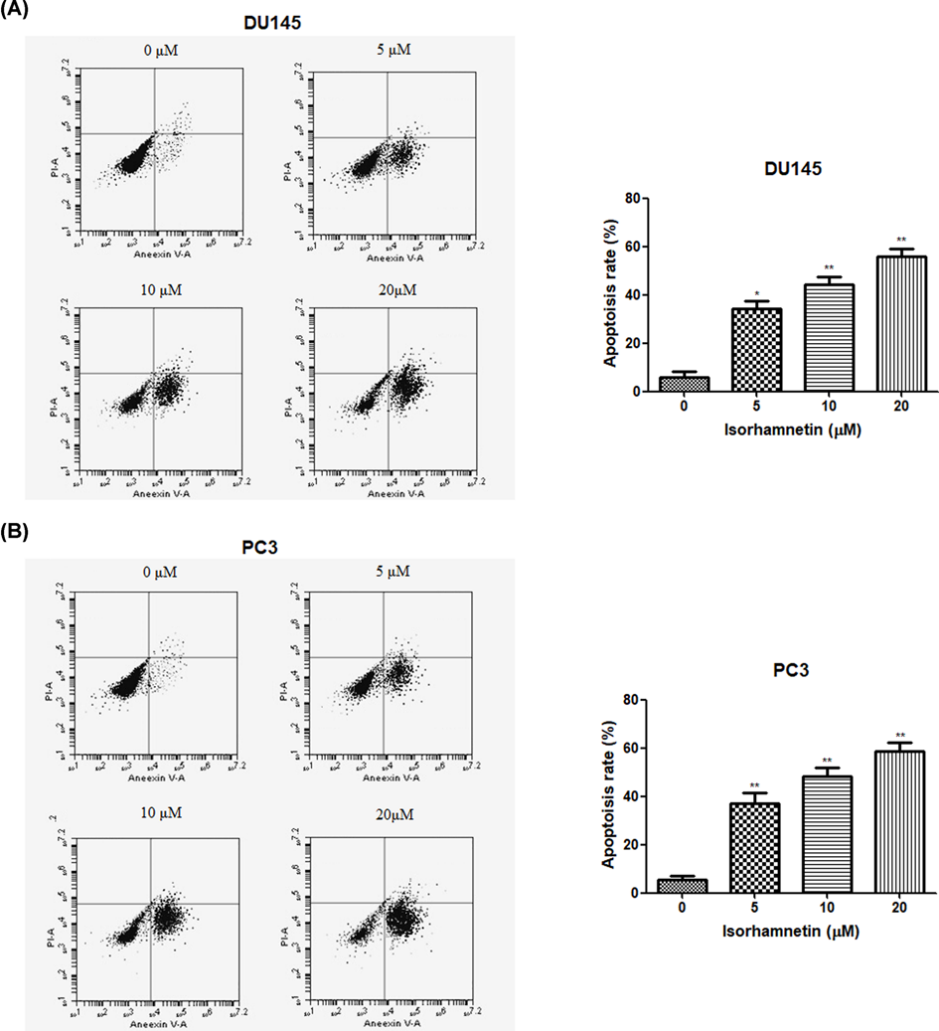
Isorhamnetin induces apoptotic cell death in prostate cancer cell lines (**A**) Effects of Isorhamnetin (5, 10, and 20 μM) on the apoptotic cell death of DU145 cells. (**B**) Effects of Isorhamnetin (5, 10, and 20 μM) on the apoptotic cell death of DU145 cells. Related data appear as the means ± SD of three separate assays. *P<0.05 and **P<0.01 vs control.

To determine whether Isorhamnetin can induce apoptotic death in androgen-independent DU145 and PC3 cells, next, both cancer cells were exposed to Isorhamnetin (5, 10, and 20 μM) for 48 h and then the apoptosis was examined by flow cytometry in treated cells double-stained with Annexin V and PI. Flow cytometry analysis showed that supplement with different concentrations of Isorhamnetin (5, 10, and 20 μM) induced a concentration-dependent apoptotic cell death in DU145 and PC3 cells (*P*<0.05; Figure 3A,B). The percentage of apoptotic cells in the cells treated with Isorhamnetin at the concentrations of 5, 10, 20 μM was 35.45, 45.60, and 55.40% for DU145 cells, and 37.78, 47.26, and 58.41% for PC3 cells, respectively, which were significantly different from those in the untreated control (*P*<0.05 or *P*<0.01). Our data definitively demonstrate Isorhamnetin could induce the apoptotic death of DU145 and PC3 cells.

**Figure 4:**
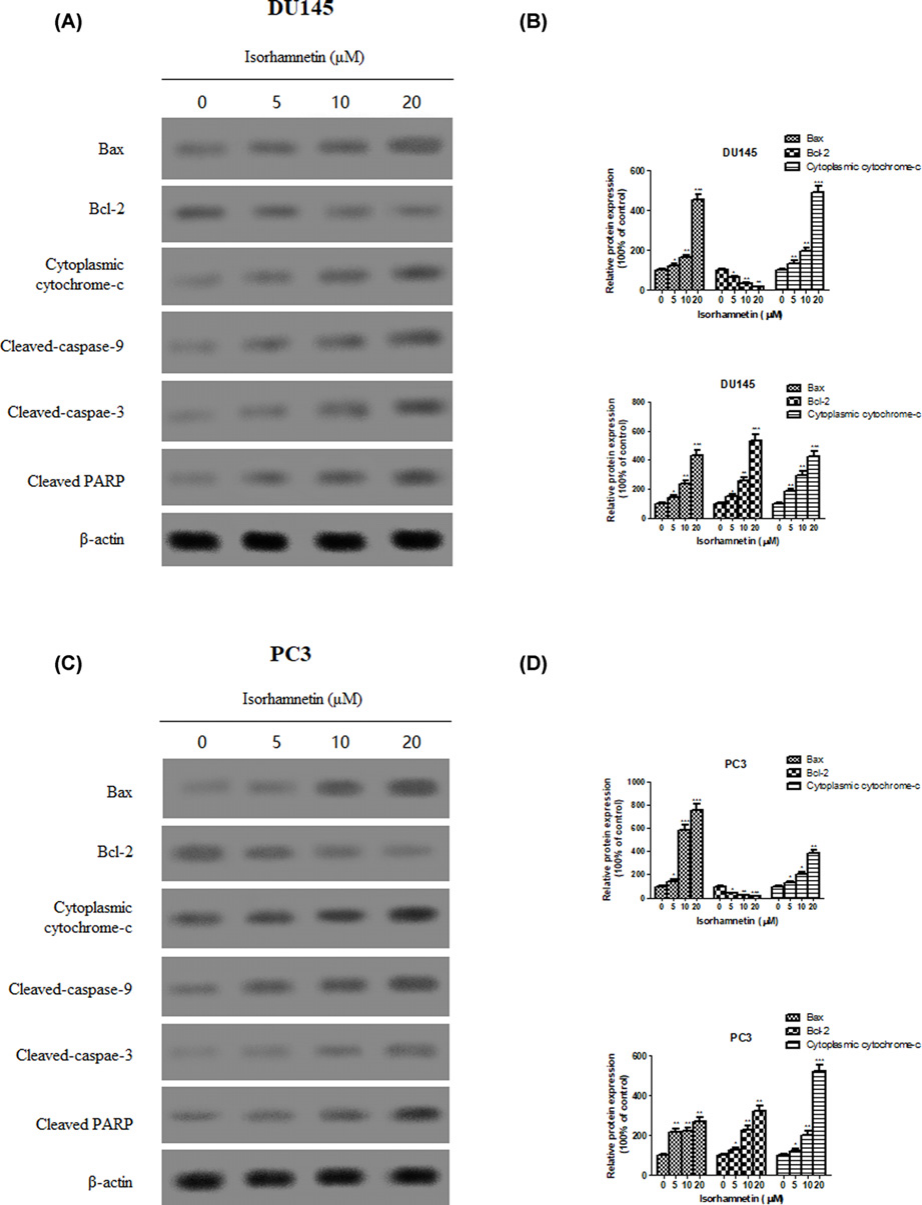
Isorhamnetin alters the expression of apoptosis-related protein in prostate cancer cell lines (**A**) Effects of Isorhamnetin (5, 10, and 20 μM) on the expression of Bax, Bcl-2, and cytoplasmic cytochrome-*c* protein in DU145 cells. (**B**) Relative levels of each protein (Bax, Bcl-2, and cytoplasmic cytochrome-*c*) compared with control (%) in DU145 cells. (**C**) Effects of Isorhamnetin (5, 10, and 20 μM) on the expression of cleaved-caspase 9, cleaved-caspase 3, and cleaved-PARP protein in PC3 cells. (**D**) Relative levels of each protein (cleaved-caspase 9, cleaved-caspase 3, and cleaved-PARP) compared with control (%) in PC3 cells. Related data appear as the means ± SD of three separate assays. *P<0.05, **P<0.01, and ***P<0.001 vs control.

Western blot examination was used to investigate changes of apoptosis-related protein expression known to possess roles in activation of signal transduction cascades. As shown in (Figure 4A–D) treatment with Isorhamnetin (5, 10, and 20 μM) markedly decreased the levels of antiapoptotic protein Bcl-2 but increased the levels of proapoptotic protein Bax and cytoplasmic cytochrome-*c* of DU145 and PC3 cells in a dose-dependent fashion. There was also a marked increase in the amounts of cleaved caspase-9 and caspase-3 in both cells treated with Isorhamnetin (5, 10, and 20 μM). Accordingly, the addition of Isorhamnetin led to cleavage of PARP. These data support the view that Isorhamnetin-induced apoptotic cell death in both androgen-independent prostate cancer cells was mediated via a mitochondria-dependent pathway.

### Isorhamnetin inhibits cell invasion/migration and alters the expression of tumor metastasis-related protein of androgen-independent DU145 and PC3 cells

**Figure 5:**
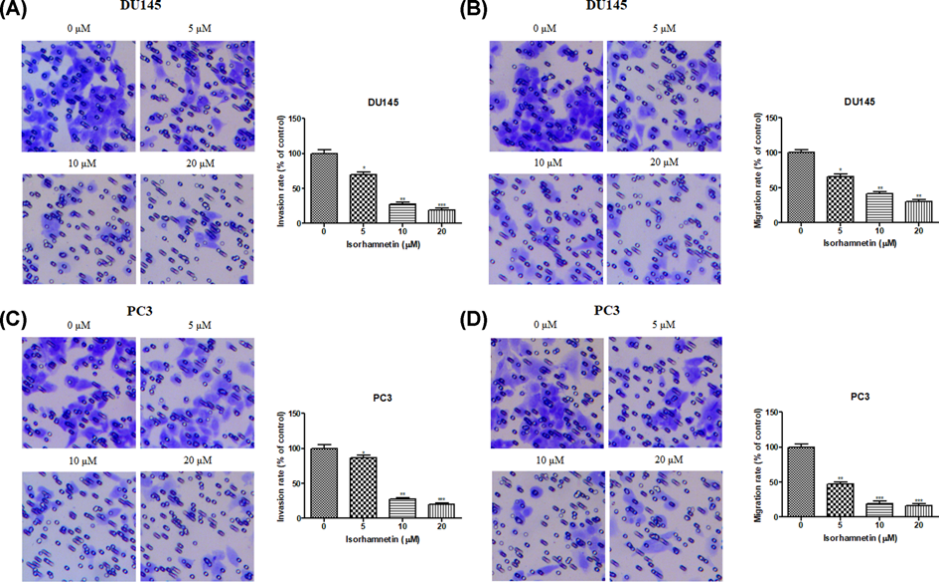
Isorhamnetin inhibits invasion and migration in prostate cancer cell lines (**A**) Effects of Isorhamnetin (5, 10, and 20 μM) on the invasion of DU145 cells. (**B**) Effects of Isorhamnetin (5, 10, and 20 μM) on the migration of DU145 cells. (**C**) Effects of Isorhamnetin (5, 10, and 20 μM) on the invasion of PC3 cells. (**D**) Effects of Isorhamnetin (5, 10, and 20 μM) on the migration of PC3 cells. Related data appear as the means ± SD of three separate assays. *P<0.05, **P<0.01, and ***P<0.001 vs control.

To examine the role of Isorhamnetin in cellular invasion and migration of prostate cancer cell, we employed Boyden chamber assay coated with Matrigel or not to respectively characterize their response to Isorhamnetin at the concentration of 5, 10, and 20 μM. As shown in Figure 5A,B, in comparison with untreated cells, Isorhamnetin treatment notably decreased the number of invaded and migrated DU145 cells in a dose-dependent manner after treatment for 48 h (*P*<0.05, *P*<0.01, or *P*<0.001). Furthermore, Isorhamnetin also reduced the invasion and migration of PC3 cells in Matrigel invasion chambers (Figure 5C,D); the effect was especially pronounced at the high dose (20 μM) as compared with the untreated cells (*P*<0.05, *P*<0.01, or *P*<0.001).

**Figure 6:**
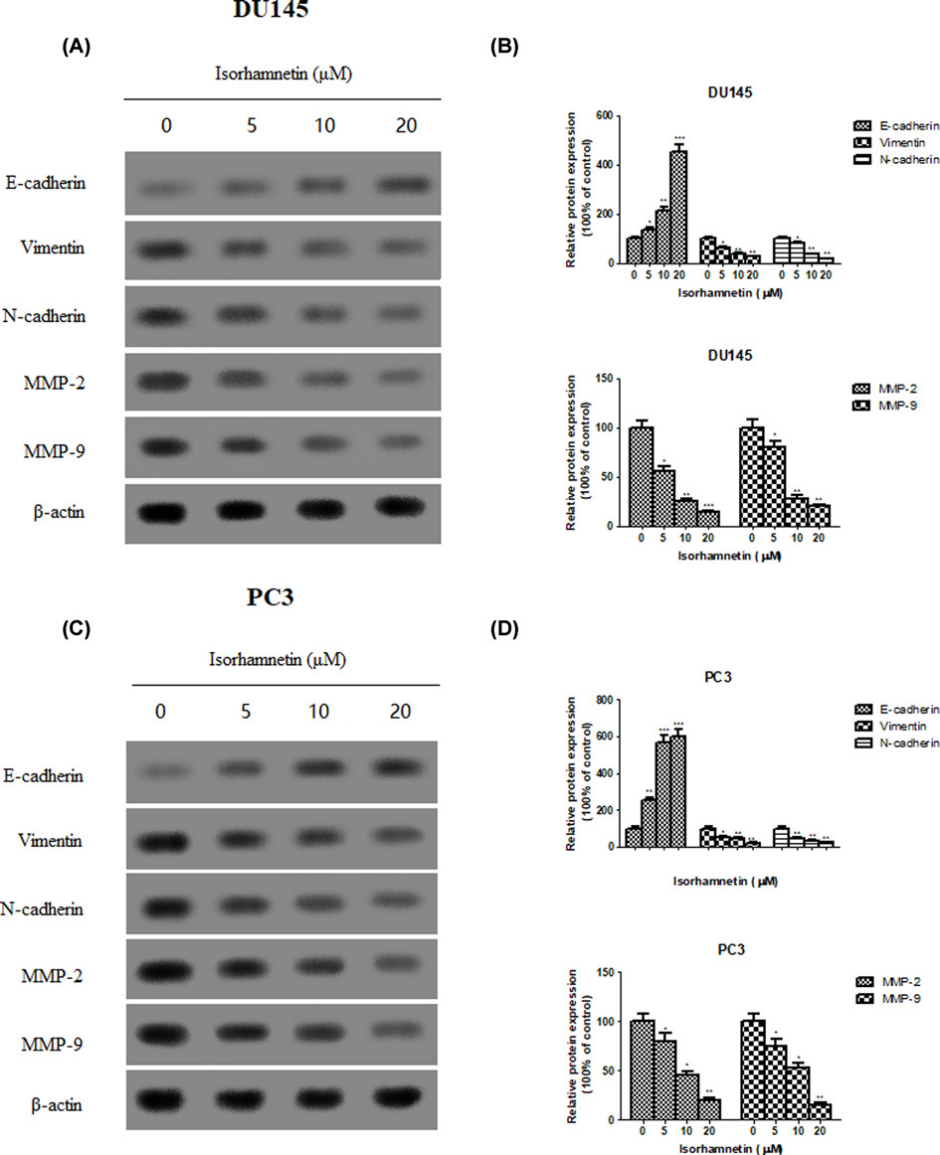
Isorhamnetin alters the expression of EMT-related protein in prostate cancer cell lines. (**A**) Effects of Isorhamnetin (5, 10, and 20 μM) on the expression of E-cadherin, Vimentin, N-cadherin, MMP-2, and MMP-9 protein in DU145 cells. (**B**) Relative levels of each protein (E-cadherin, Vimentin, N-cadherin, MMP-2, and MMP-9) compared with control (%) in DU145 cells. (**C**) Effects of Isorhamnetin (5, 10, and 20 μM) on the expression of E-cadherin, Vimentin, N-cadherin, MMP-2, and MMP-9 protein in PC3 cells. (**D**) Relative levels of each protein (E-cadherin, Vimentin, N-cadherin, MMP-2, and MMP-9) compared with control (%) in PC3 cells. Related data appear as the means ± SD of three separate assays. *P<0.05, **P<0.01, and ***P<0.001 vs control.

To further elucidate the mechanisms underlying the anti-metastatic effects of Isorhamnetin, the expression of several epithelial−mesenchymal transition (EMT) marker (E-cadherin, vimentin, and N-cadherin) and androgen-independent prostate cancer-associated proteins (MMP-2 and MMP-9) were assessed by Western blot analysis. Isorhamnetin treatment increased the protein expression of epithelial marker E-cadherin, but decreased the protein expression of mesenchymal markers vimentin and N-cadherin, as well as MMP-2 and MMP-9 (Figure 6A–D).

### Isorhamnetin inhibits the activation of PI3K/Akt/mTOR pathways and alters expression of downstream regulators of apoptosis

**Figure 7:**
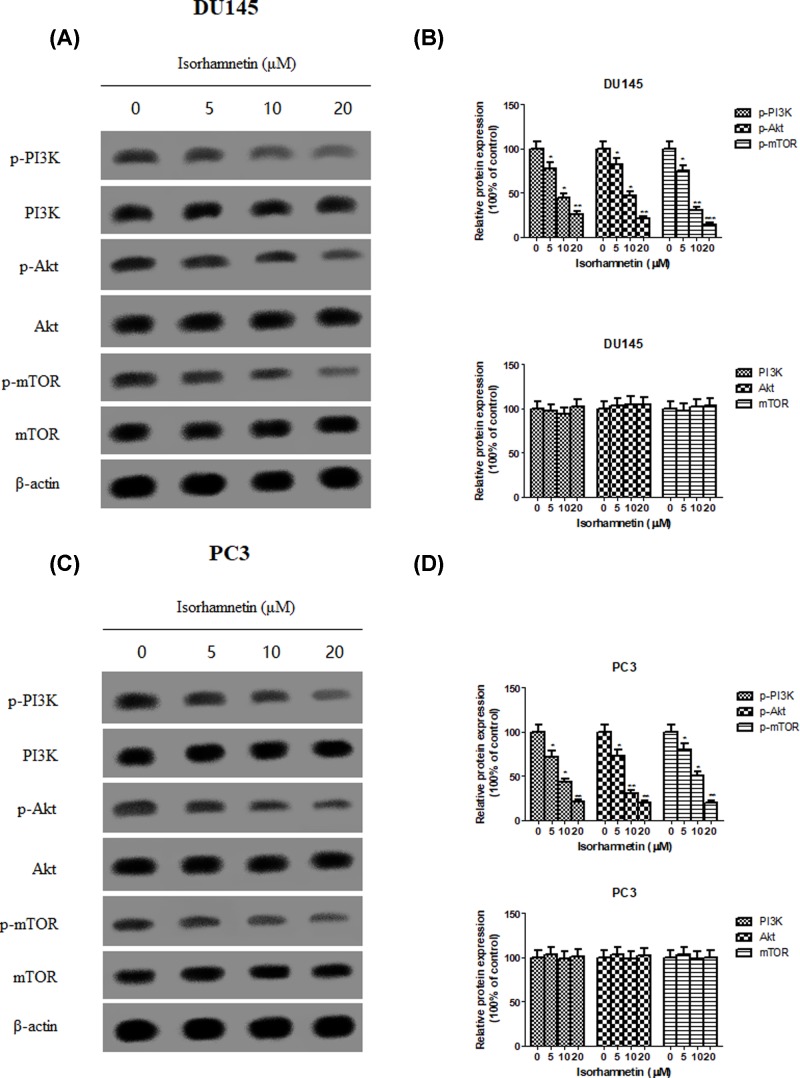
Isorhamnetin inhibits the phosphorylation expression of PI3K, Akt and mTOR protein in prostate cancer cell lines (**A**) Effects of Isorhamnetin (5, 10, and 20 μM) on the expression of p-PI3K, PI3K, p-Akt, Akt, p-mTOR, and mTOR protein in DU145 cells. (**B**) Relative levels of each protein (p-PI3K, PI3K, p-Akt, Akt, p-mTOR, and mTOR) compared with control (%) in DU145 cells. (**C**) Effects of Isorhamnetin (5, 10, and 20 μM) on the expression of p-PI3K, PI3K, p-Akt, Akt, p-mTOR, and mTOR protein in PC3 cells. (**D**) Relative levels of each protein (p-PI3K, PI3K, p-Akt, Akt, p-mTOR, and mTOR) compared with control (%) in PC3 cells. Related data appear as the means ± SD of three separate assays. *P<0.05, **P<0.01, and ***P<0.001 vs control.

To investigate the upstream pathway of Isorhamnetin on apoptosis and metastasis, the expression level of PI3K, Akt, and mTOR and their phosphorylated forms was measured by Western blot analysis. Western blot analysis showed that the phosphorylation levels of PI3K, Akt, and mTOR were dose-dependently inhibited in Isorhamnetin-treated DU145 cells as compared with those in vehicle-treated cells (Figure 7A,B), whereas total PI3K, Akt, and mTOR protein expression remained unchanged. The same tendency was observed in PC3 cells following Isorhamnetin treatment at three concentrations (Figure 7C,D).

**Figure 8:**
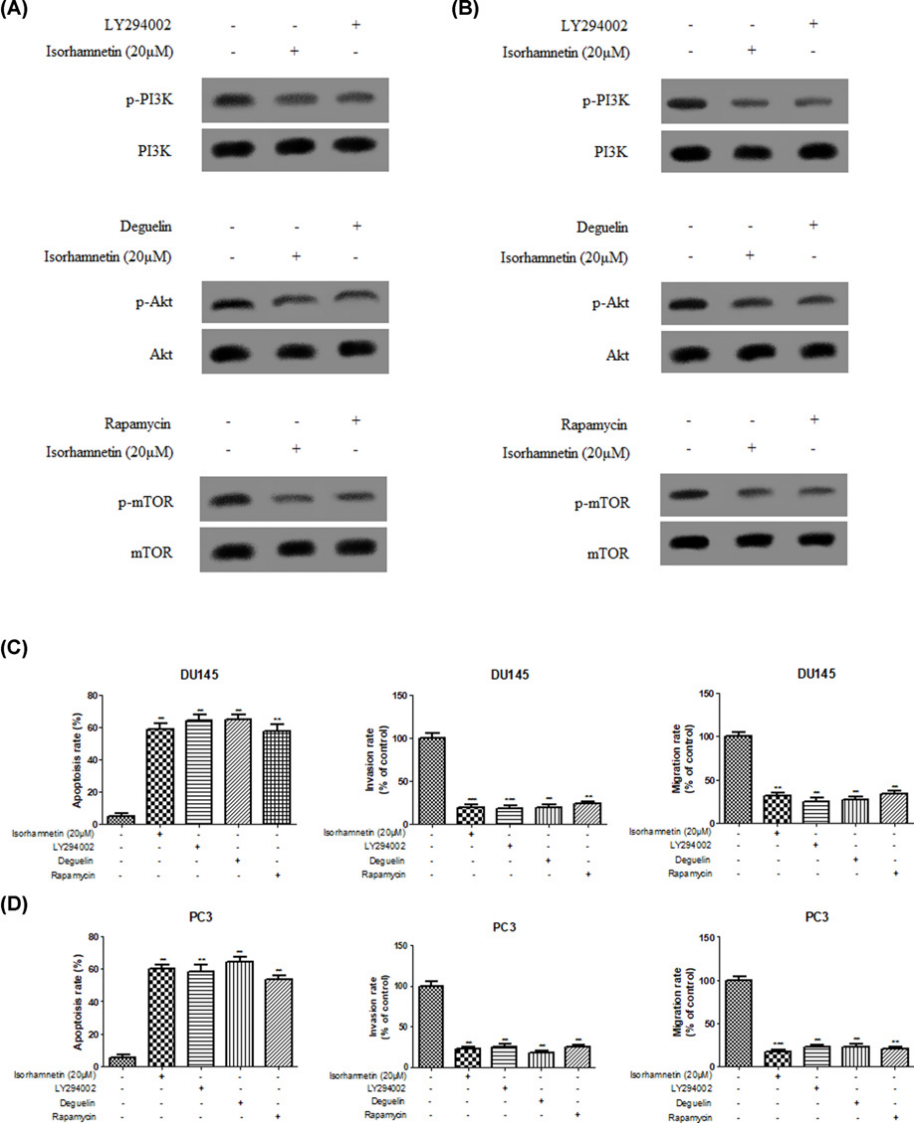
PI3K/Akt/mTOR pathway inhibitor (LY294002/deguelin/rapamycin) or Isorhamnetin (20 μM) treatment inhibits the phosphorylation expression of PI3K, Akt and mTOR protein, induces apoptotic cell death and inhibits invasion and migration in prostate cancer cell lines (**A**) Effects of Isorhamnetin (20 μM) or LY294002/deguelin/rapamycin on the expression of p-PI3K, PI3K, p-Akt, Akt, p-mTOR, and mTOR protein in DU145 cells. (**B**) Effects of Isorhamnetin (20 μM) or LY294002/deguelin/rapamycin on the expression of p-PI3K, PI3K, p-Akt, Akt, p-mTOR, and mTOR protein in PC3 cells. (**C**) Effects of Isorhamnetin (20 μM) or LY294002/deguelin/rapamycin on the apoptotic cell death, invasion, and migration of DU145 cells. (**D**) Effects of Isorhamnetin (20 μM) or LY294002/deguelin/rapamycin on the apoptotic cell death, invasion, and migration of DU145 cells. Related data appear as the means ± SD of three separate assays. *P<0.05, **P<0.01, and ***P<0.001 vs control.

To further explore the role of PI3K/Akt/mTOR signaling pathways on the regulation of apoptosis and anti-metastasis in prostate cancer cells in response to Isorhamnetin treatment, both DU145 and PC3 cells were pretreated with PI3K/Akt/mTOR pathway inhibitors (LY294002/deguelin/rapamycin) or Isorhamnetin (20 μM) for 48 h. As shown in Figure 8A,B, decreased protein expression of p-PI3K, p-Akt, and p-mTOR was observed in DU145 and PC3 cells following respective LY294002/deguelin/rapamycin or Isorhamnetin (20 μM) treatment, but PI3K, Akt, and mTOR protein remain unchanged. Of note, Flow cytometry and Transwell analysis (Figure 8C,D) also showed that Isorhamnetin (20 μM) or LY294002/deguelin/rapamycin could remarkably induce an increase in the percentage of apoptotic cells and inhibit invasion and migration ability of both prostate cancer cells *in vitro* when compared with the control cells (*P*<0.05, *P*<0.01, or *P*<0.001).

## Discussion and conclusions

In recent decades, biologically active derivatives from medicinal plants have attracted extensive attention in cancer therapy because of their promising efficacy and less side effects [21]. Isorhamnetin has been recognized as a potential antitumor medicine in several types of cancer, including esophageal and gastric cancer, skin, leukemia, lung, and colon cancer. However, the antitumor effect of Isorhamnetin on androgen-independent prostate cancer cells remains pooly unknown. To this end, the present study first conducted an MTT assay to explore the anticancer activity of Isorhamnetin in androgen-independent (DU145 and PC3) and androgen-dependent prostate cell line (LNCaP). Interestingly, Isorhamnetin can be selectively cytotoxic to androgen-independent DU145 and PC3 cells in a concentration- (0.5–20 μM) and time-dependent (24, 48, and 72 h) manner, and exerted no toxicity effect on normal human prostate epithelial PrEC cells under the same conditions. Then we decided to use the following optimal factors (concentration: 5, 10, and 20 μM; time: 48 h; cell lines: DU145 and PC3) in the following experiments to decipher deep mechanistic insights into the cytotoxic activity of Isorhamnetin on androgen-independent prostate cancer cell types and identify the key signaling molecules responsible for this effect. The extracellular LDH release is a feature of necrotic death [22]. Consistent with MTT assay, the LDH release was increased in the culture surpernatant of androgen-independent DU145 and PC3 cells following Isorhamnetin (5, 10, and 20 μM) treatment for 48 h in a concentration-dependent manner, which was obviously significant from the control group (*P*<0.05 or 0.01). No obvious LDH release was observed in human prostate epithelial PrEC cells and only a slight LDH increase occurred in androgen-dependent LNCaP cells in response to Isorhamnetin (20 μM) treatment (*P*<0.05). These observations suggested that Isorhamnetin treatment can selectively cause cell death of androgen-independent DU145 and PC3 cells.

Apoptosis, namely programmed cell death, is considered as an important mechanism in the inhibition of cancer cells by many anticancer agents [23,24]. In this study, the significant cell loss demonstrated by MTT assay encourages further study on the proapoptotic effects of Isorhamnetin in androgen-independent prostate cancer cells. To further substantiate whether the cytotoxicity of Isorhamnetin is mediated by the induction of apoptosis, DU145, and PC3 cells were stained with Annexin V–FITC and PI, and subsequently analyzed by flow cytometry. The present work confirmed that the exposure of DU145 and PC3 cells to Isorhamnetin (5, 10, 20 μM) resulted in a dose-dependent increase in the apoptotic cell population, suggesting that Isorhamnetin could induce apoptosis in both prostate cancer cells. To further explore more insight into the apoptotic induction mechanism of Isorhamnetin, we analyzed several important apoptotic signaling proteins (Bax, Bcl-2. caspase-3, caspase-8, caspase-9, and cytochrome-*c*) in DU145 and PC3 cells after Isorhamnetin treatment for 48 h. Upon the mitochondrial trans-membrane potential is damaged due to the alteration in the balance between the antiapoptotic (e.g. Bcl-2) and proapoptotic (e.g. Bax) Bcl-2 family members, the cytochrome-*c* would be released into the cytoplasm from the mitochondria that in turn leads to activation of caspases-9 and -3, as well as the subsequent cleavage of cellular substrates PARP, thus initiating the mitochondrial pathway of apoptosis [25–27]. Western blot analysis demonstrated that Isorhamnetin (5, 10, and 20 μM) could directly elevate the expression of Bax, cytoplasmic cytochrome-*c*, cleaved caspase-9, cleaved caspase-3, and cleaved PARP protein, but decreased the level of Bcl-2 protein. These results indicate that a mitochondrion-dependent intrinsic apoptotic pathway may be one of the major apoptosis pathways triggered by Isorhamnetin in DU145 and PC3 cells.

Cell movement is one of the most critical and primary factors associated with cancer therapeutic efficacy and prognostic survival [28]. Considering the high aggressive metastasis property of androgen-independent prostate cancer, discovery of antitumor agents that target the blocking potential of cancer cells proliferation and metastasis would be one of attractive strategies for this disease. In the present study, the inhibitory effect of Isorhamnetin on cell invasion and migration was observed in both cell lines as determined by Transwell migration assay and exhibited a concentration-dependent manner. This drug was also capable of reversing the molecular changes associated with EMT, which is a crucial mechanism governing cancer cell migration and invasion [29], as showed by the increased E-cadherin (an epithelial marker) expression and decreased vimentin and N-cadherin (a mesenchymal marker) expression. This anti-metastatic effect mediated by Isorhamnetin was also accompanied by decreased protein expression of MMP-2 and MMP-9. Moreover, it has been reported that over-expression of MMPs, such as MMP-2 and MMP-9, can enhance cancer cells’ ability to metastasize including androgen-independent prostate cancer [30,31]. These *in vitro* findings further provided molecular evidence supporting the inhibitory effects of Isorhamnetin on the invasion and migration of androgen-independent prostate cancer DU145 and PC3 cells.

Apart from the involvement of above mechanism in Isorhamnetin-induced antitumor activity, abnormal activation of the PI3K/Akt/mTOR pathway is implicated in both the pathogenesis of prostate cancer and the development of resistance to anticancer therapies [32,33], suggesting that targeted inhibition of individual components in this pathway may be a potential strategy for cancer therapy [34]. Therefore, we examined whether Isorhamnetin inhibits activation of this pathway in DU145 and PC3 cancer cells. Next, the phosphorylation of key factors involved in the PI3K/AKT/mTOR pathway was assessed by Western blot. The results showed that the protein levels of p-PI3K, p-AKT, and p-mTOR was remarkably down-regulated in both DU145 and PC3 cells treated with Isorhamnetin (5, 10, and 20 μM) for 48 h when compared with the vehicle group. To further confirm the inhibitory role of the PI3K/Akt/mTOR signaling pathway involved in antitumor effect of Isorhamnetin in both cancer cells, we assessed the PI3K/Akt/mTOR pathway inhibitors (LY294002/deguelin/rapamycin) on the regulation of cell apoptosis and metastasis. The same results occurred in DU145 and PC3 cells following LY294002/deguelin/rapamycin or single Isorhamnetin treatment. These results suggest that treatment with Isorhamnetin could significantly inhibit PI3K/Akt/mTOR signaling, thus generating highlighted anticancer effects.

Taken together, the findings of the present study show that the selective antitumor activity of Isorhamnetin are attributed to the induction of apoptosis and the blockade of migration and invasion, which might be mediated through inducing a mitochondrion-dependent intrinsic apoptotic pathway and inhibiting EMT and the PI3K/Akt/mTOR signaling. The results of this trial will provide an indication of the feasibility of using Isorhamnetin as cancer therapeutics for androgen-independent prostate cancer treatment. However, the promising anticancer mechanism of Isorhamnetin should be further studied in animal models of androgen-independent prostate cancer.

## Abbreviations

DMEM: Dulbecco’s modified Eagle’s medium
ECL: enhanced chemiluminescence
EMT: epithelial–mesenchymal transition
FBS: fetal bovine serum
FITC: fluorescein isothiocyanate
LDH: lactate dehydrogenase
MMP: matrix metalloproteinase
MTT: 3-(4,5-dimethylthiazol-2-yl)-2,5-diphenyltetrazolium bromide
OD: optical density
PI: Propidium Iodide
p-Akt: phosphorylated Akt
p-mTOR: phosphorylated mTOR
p-P13K: phosphorylated PI3K
